# Assessment of Tongue Strength in Sarcopenia and Sarcopenic Dysphagia: A Systematic Review and Meta-Analysis

**DOI:** 10.3389/fnut.2021.684840

**Published:** 2021-06-24

**Authors:** Kuan-Cheng Chen, Tsung-Min Lee, Wei-Ting Wu, Tyng-Guey Wang, Der-Sheng Han, Ke-Vin Chang

**Affiliations:** ^1^Department of Physical Medicine and Rehabilitation, National Taiwan University Hospital and National Taiwan University College of Medicine, Taipei, Taiwan; ^2^Department of Physical Medicine and Rehabilitation, National Taiwan University Hospital, Bei-Hu Branch, Taipei, Taiwan; ^3^Center for Regional Anesthesia and Pain Medicine, Wang-Fang Hospital, Taipei Medical University, Taipei, Taiwan

**Keywords:** sarcopenia, dysphagia, tongue strength, frailty, tongue pressure

## Abstract

Sarcopenic dysphagia is defined as difficulty in swallowing due to sarcopenia, which may be related to weakness of the tongue muscles. This meta-analysis aimed to explore the association between tongue strength and sarcopenia and to determine whether tongue strength measurement could be a specific indicator of sarcopenic dysphagia. We conducted a systematic search of electronic databases from their inception to February 2021 for clinical studies that investigated tongue strength in participants with and without sarcopenia. The primary outcome was the weighted mean difference (WMD) and standardized mean difference (SMD) of tongue pressure between the different groups. The secondary outcome was the correlation of tongue pressure with the subcomponents that defined sarcopenia. Ten studies that involved 1,513 participants were included in the meta-analysis. Compared with those without sarcopenia, patients with sarcopenia had significantly less tongue pressure, with a WMD of −4.353 kPa (95% CI, −7.257 to −1.450) and an SMD of −0.581 (95% CI, −0.715 to −0.446). There was no significant difference in tongue pressure between patients with sarcopenic dysphagia and those with non-sarcopenic dysphagia, with a WMD of −1.262 kPa (95% CI, −8.442 to 5.918) and an SMD of −0.187 (95% CI, −1.059 to 0.686). Significant positive associations were identified between tongue pressure and grip strength and between tongue pressure and gait speed, with correlation coefficients of 0.396 (95% CI, 0.191 to 0.567) and 0.269 (95% CI, 0.015 to 0.490), respectively. Reduced tongue strength is associated with sarcopenia but is not an exclusive marker for sarcopenic dysphagia. Tongue strength correlates with the values of subcomponents that define sarcopenia. In patients with low performance of sarcopenia subcomponent, tongue pressure must be examined to diagnose subclinical dysphagia.

**Protocol registration:** This meta-analysis was registered on INPLASY (registration number INPLASY202120060).

## Introduction

Sarcopenia was first used by Rosenberg to describe an age-related decrease in muscle mass (1). According to the European Working Group on Sarcopenia in Older People (EWGSOP) (2) and the Asian Working Group for Sarcopenia (AWGS) (3) diagnostic criteria, sarcopenia is defined as low muscle mass, strength, and/or physical performance. The prevalence of sarcopenia has been reported to be between 1 and 29% in the community-dwelling population and between 14 and 33% in residents living in long-term care facilities (4). The association between sarcopenia and adverse health outcomes, such as mortality, incidence of falls, and longer hospitalization, has been reported in previous studies (5, 6). In addition, studies have shown that sarcopenia not only reduces the strength of limbs but also that of the oropharyngeal muscles, leading to swallowing impairment (7, 8).

Dysphagia is a term derived from Greek words, meaning worsening in eating (9) and is related to organic or neurological diseases, such as nasopharyngeal cancer, stroke, Parkinson's disease, and dementia (10). Sarcopenic dysphagia is characterized by sarcopenia of the entire body and swallowing-related muscles (11). The swallowing process can be divided into four phases: oral preparatory, oral, pharyngeal, and esophageal phases. The tongue plays a key role in bolus transport from the oral cavity to the pharynx. Tongue movements stimulate oropharyngeal receptors and trigger subsequent swallowing events (12). Abnormal tongue function is associated with oral and pharyngeal dysphagia (13). It has been reported that tongue strength is positively correlated with swallowing function (14). Aging-related fatty infiltration, amyloid deposition, and loss of tongue muscle fibers can lead to a decrease in tongue pressure (15). In addition, decreased tongue pressure during swallowing has been observed in patients with post-stroke dysphagia (16).

The diagnosis of sarcopenic dysphagia is important because sarcopenic dysphagia increases the risk of complications such as dehydration, malnutrition, and aspiration pneumonia (17). The prevalence of dysphagia in the sarcopenic population was reported to be 32% (18). Tongue strength measurement has been proposed as a diagnostic tool for sarcopenic dysphagia (19). The modified water swallowing test (MWST) has been widely used by medical practitioners to screen for dysphagia (20). However, it puts the examinees at risk of choking. The measurement of tongue strength is theoretically safer and more reliable than MWST. Since the measurement of tongue pressure is an objective method for assessing tongue strength, whether or not tongue pressure differs in the sarcopenic population is a clinically important issue. Therefore, the purpose of the meta-analysis was two-fold: (1) to explore the association between tongue strength and sarcopenia and (2) to determine whether tongue strength measurement could be a specific indicator for sarcopenic dysphagia.

## Methods

### Protocol Registration

The study was performed in accordance with the guidelines of the Preferred Reporting Items for Systematic Reviews and Meta-Analyses (PRISMA) program (21). The meta-analysis was prospectively registered on Inplasy.com (INPLASY202120060).

### Studies Search and Selection

PubMed (US National Library of Medicine) and Embase (Wolters Kluwer Ovid) were searched for cross-sectional, case-control, and cohort studies that investigated tongue strength in the sarcopenia population from their inception to February 2021. Key search terms included: “sarcopenia,” “frailty,” “dysphagia,” “swallowing disorder,” “tongue pressure,” “tongue strength” (Appendix 1). There was no restriction on language during the literature search. Furthermore, relevant narratives and systemic reviews were manually retrieved for potentially eligible articles.

### Inclusion and Exclusion Criteria

Studies were included if they: (1) investigated human subjects over the age of 18 years; (2) provided measurements for tongue pressure; (3) provided how sarcopenia was evaluated and (4) evaluated swallowing performance. The study types were divided into cross-sectional studies, cohort studies, case-control studies, and clinical trials.

The following studies were excluded: (1) case reports, case series, and research protocols; (2) studies that did not measure tongue pressure and sarcopenia components; (3) studies that validated technologies or devices for tongue strength assessment; and (4) studies that lacked a control group with normal muscle volume and function.

### Quality Assessment

The Newcastle–Ottawa Scale (NOS) was used to assess the quality of each study (22). It evaluates eight aspects of each retrieved study: representativeness of sarcopenic patients, selection of control, ascertain of tongue pressure measurement, outcome of interest not present at start, comparability of cohorts, assessment of outcome, enough follow-up period, and adequacy of follow-up. The quality assessment was conducted by both reviewers individually, while the outcomes of the evaluation were decided based on a consensus or by the corresponding author.

### Data Extraction

Two authors (K.C.C and K.V.C) independently screened the titles and abstracts to determine whether the articles met the scope of the present meta-analysis. The full texts of the pertinent articles were retrieved for further data extraction. The author, publication year, study design, diagnostic criteria for sarcopenia, number of included patients, population characteristics, sex ratio, and data collection period were extracted from all included studies. If some data were missing in the published articles, the corresponding authors of the original studies were contacted for the required information. Questions arising from data abstraction were resolved through discussions or by the corresponding author.

### Statistical Analysis

The primary outcome included the weighted mean difference (WMD) and standardized mean difference (SMD) between the groups. The SMD was calculated as the difference in the mean tongue pressure divided by the pooled standard deviation (23). The WMD provided the absolute between-group difference in tongue pressure in kPa, whereas the SMD facilitated the awareness of the magnitude of the effect regarding tongue strength discrepancy for the two target populations. An SMD of 0.2, 0.5, and 0.8, is considered a small, moderate, and large effect size, respectively (24). The secondary outcome was the correlation of tongue pressure with the subcomponents of sarcopenia. The correlation coefficients were analyzed using the Hedges-Olkin method based on the Fisher Z transformation of the variables (25). We also analyzed the association between sarcopenia and low tongue pressure using the risk ratio (26).

The random effect model was used for pooling the data, considering the variations in the study designs and enrolled participants. The between-group heterogeneity was evaluated using the Cochrane's Q and *I*^2^ statistics. An *I*^2^ > 50% was considered to indicate significant heterogeneity (27). Publication bias was determined by visual inspection of the funnel plots and the p-value of the Egger's test (28). All statistical analyses were conducted using Comprehensive Meta-analysis Software v 3 (Biostat, Englewood, NJ), and a *p* < 0.05 was considered to indicate statistical significance.

## Results

### Literature Search

**Figure 1:**
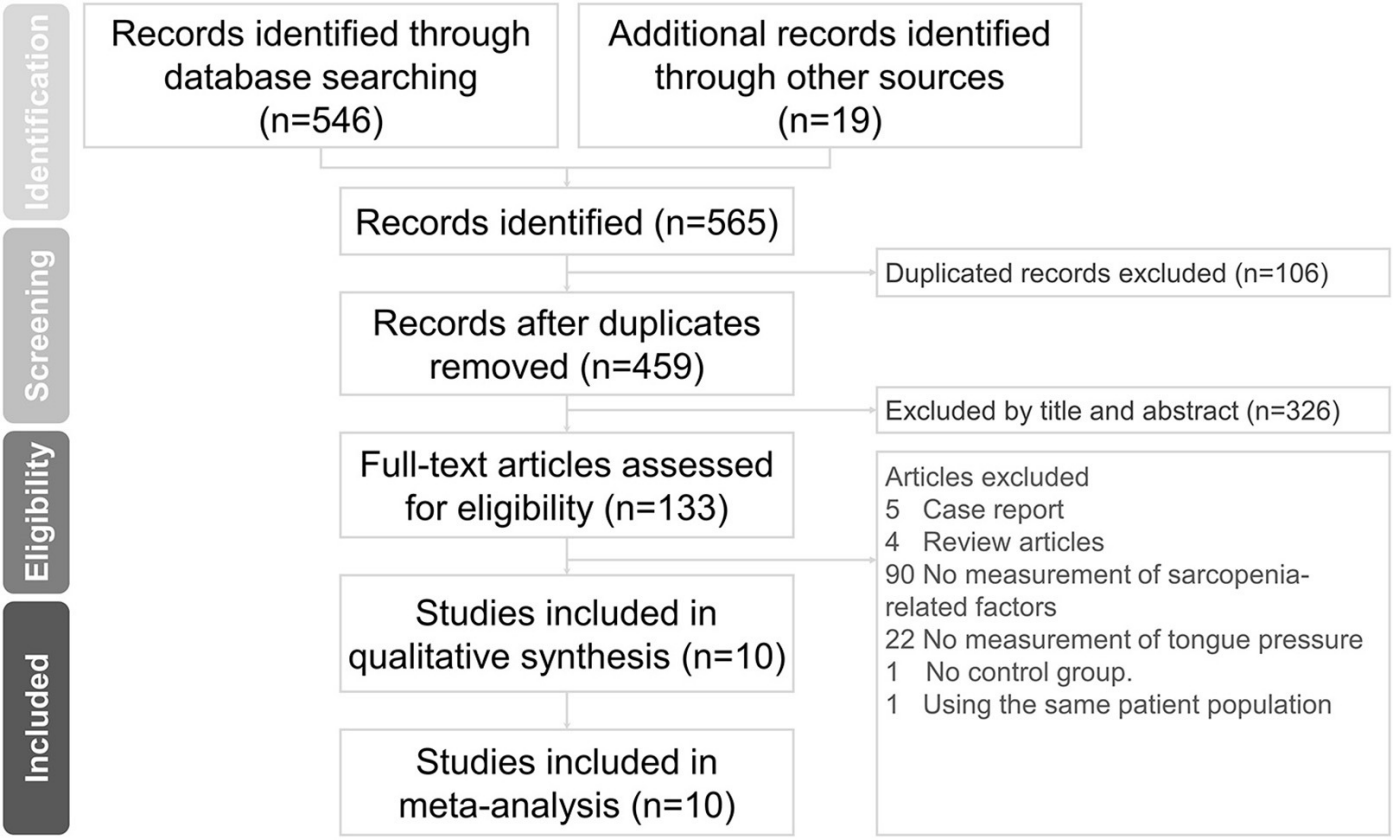
Preferred Reporting Items for Systematic Reviews and Meta-Analyses (PRISMA) flow diagram for the study.

The initial literature search identified 565 articles. After excluding 106 duplicate articles and 326 non-relevant articles by screening their titles and abstracts, 133 studies were eligible for subsequent evaluation. Five case reports, four review articles, 90 studies that did not measure sarcopenia-related factors, 22 studies that did not assess tongue pressure, one study without a control group, and one study (29) involving the same patient cohort as another study were excluded (Figure 1). Finally, a total of ten articles were included in the meta-analysis (8, 18, 30–37). These articles comprised of nine cross-sectional studies (8, 30–37) and one cohort study (18). Data on the number of sarcopenic and non-sarcopenic groups were missing in one study and were retrieved by contacting the corresponding author of the article (36). The details of the included studies are presented in Table 1.

**Table 1:** Characteristics of the included study.

**References**	**Study design**	**Patient characteristic**	**Outcome measurement**	***N* Sarcopenia**	***N* non-sarcopenic**	**Age**	**Sex ratio M/F**	**Data collection period**	**Swallowing evaluation tool**	**Country**
Shimizu et al. (37)	Cross-sectional	Admissions for orthopedic conditions, aged ≥ 65 years, no history of cerebrovascular or neuromuscular disease, without an implanted pacemaker	TP, MNA-SF, BMI, FIM	105	92	81.3 ± 7.6	39/158	November 2018 to September 2019.	FOIS MASA	Japan
Chen et al. (35)	Cross-sectional	Elderly sarcopenic patients without dysphagia, age ≥ 65 years, living independently, fully cooperative, eat orally	TP, Submental ultrasonography,100-mL WST	47	47	75.1 ± 5.8	26/68	NA	EAT-10	Taiwan
Kobuchi et al. (36)	Cross-sectional	Patients living in nursing homes or university hospitals	TP, BMI, oral examination, BI, MNA-SF, cross-sectional area of the geniohyoid muscle, oral diadochokinesis	18	36	78.8 ± 7.1	16/38	NA	EAT-10	Japan
Sakai et al. (34)	Cross-sectional	age > 65 years, post-acute phase of illness hospitalized for rehabilitation, MMSE ≥ 21, presence of all upper and lower central incisors	TP, 100-mL WST, swallowing time, swallowing speed, lip force, MMSE, CCI, MNA-SF	86	159	84.0 (79–88)^*^	79/166	April 2015 to October 2016	FOIS	Japan
Wakabayashi et al. (18)	Prospective cohort	age > 65 years, dysphagia, referred for speech therapy	TP, BI, GNRI, BMI, total energy intake, C-reactive protein	35	73	76 ± 7	72/36	August 2016 to March 2018	FILS	Japan
Kaji et al. (33)	Cross-sectional	Type 2 diabetes, age ≥ 60 years, tolerate standing position	TP, smoking, exercise, hemoglobin A1c	17	127	71.4 ± 6.7	82/62	April 2017 to October 2017	Nil	Japan
Suzuki et al. (32)	Cross-sectional	Community-dwelling older women, age ≥65 years, walk independently, absence of dysphagia	TP, oral diadochokinesis, BMI	29	216	81.0 (75.0–85.0)^*^	NA	NA	EAT-10	Japan
Ogawa et al. (8)	Cross-sectional	Acute care hospitals or convalescent rehabilitation hospitals or long-term care hospitals or nursing homes, age> 65 years, able to answer a questionnaire	TP, thickness and area of the tongue and geniohyoid muscles, MNA-SF, BMI	36	19	82.1 ± 7.4	31/24	October 2016 to April 2017	FILS	Japan
Machida et al. (30)	Cross-sectional	Community-dwelling older adults, living independently	TP, MNA-SF, jaw-opening force, BI	68	129	78.5 ± 6.7(M) 77.8 ± 6.2(F)	97/100	NA	EAT-10	Japan
Sakai et al. (31)	Cross-sectional	age ≥65 years, post-acute phase of illness, living independently, no history of dysphagia, MMSE ≥ 21, presence of upper and lower central incisors	TP, BI, MNA-SF, BMI, serum albumin levels, CONUT, modified WST	134	40	84 (80–89)^*^	64/110	October 2014 to December 2015	FOIS EAT-10	Japan

*WST, water swallowing test; EAT, Eating assessment tool; TP, tongue pressure; BI, Barthel Index; MNA-SF, Mini Nutritional Assessment-Short Form; MMSE, Mini Mental State Examination; CCI, Charlson Comorbidity Index; FILS, Food Intake Level Scales; GNRI, Geriatric Nutritional Risk Index; BMI, body mass index; FOIS, functional oral intake scale; EAT-10, 10-item Eating Assessment Tool; CONUT, controlling nutritional status; FIM, Functional Independence Measure; MASA, Mann Assessment of Swallowing Ability; NA, not available;*

* *Interquartile range (IQR)*.

### Study Characteristics

The ten studies involved 1,513 participants, with the mean (or median) ages ranging from 71.4 to 84.0 years. Three studies recruited community-dwelling older adults (30, 32, 35), three recruited hospitalized older people (18, 31, 34), one recruited elderly patients with type 2 diabetes (33), one recruited older patients admitted for orthopedic conditions (37) and two recruited older adults who required rehabilitation (8, 33). Regarding the diagnostic algorithm for sarcopenia, one study employed the EWGSOP guidelines (31) and nine employed the AWGS criteria (8, 18, 30, 32–37). The diagnostic tools and criteria for sarcopenia in the included studies are shown in Table 2. The tools used for the evaluation of swallowing function are summarized in Table 1. They include the 10-item Eating Assessment Tool, Functional Oral Intake Scale, Food Intake Level Scale, and MWST and Mann Assessment of Swallowing Ability.

**Table 2:** Diagnostic tools and criteria of sarcopenia in the included studies.

**References**	**Muscle strength**	**Muscle volume**	**Muscle function**	**Diagnostic algorithm**
	**Cut-off points**			
Shimizu et al. (37)	Jamar digital handgrip gauge (MG-4800; CHARDER Electronic, Taichung, Taiwan)	BIA	NA	AWGS: low HGS + low SMI
	<28 kg for male, <18 kg for female	②	NA	
Chen et al. (35)	Handheld dynamometer	DEXA/BIA	5-m walk test	AWGS: low HGS + low SMI ± low gait speed
	①	②/③	④	
Kobuchi et al. (36)	Handgrip dynamometer (Takei Scientific Instruments Co., Ltd).	BIA	5-m walk test in a 9 m path	AWGS: low SMI + low HGS or low gait speed
	①	③	④	
Sakai et al. (34)	Digital grip strength dynamometer	CC	NA	AWGS: low HGS + low CC
	①	<34 cm for male; <33 cm for female	NA	
Wakabayashi et al. (18)	NA	CC	NA	AWGS: low HGS + low CC ± low gait speed
	①	<30 cm for male; <29 cm for female	④	
Kaji et al. (33)	Handgrip dynamometer (Smedley; Takei Scientific Instruments, Niigata, Japan)	BIA	NA	AWGS: low HGS + low SMI
	①	②	NA	
Suzuki et al. (32)	Handgrip dynamometer (TTM, Tokyo, Japan)	BIA	5-m walk test	AWGS: low HGS + low SMI ± low gait speed
	①	②	④	
Ogawa et al. (8)	Grip strength	CC	NA	AWGS: low HGS + low CC ± low gait speed
	①	<34 cm for male; <33 cm for female	④	
Machida et al. (30)	Handgrip dynamometer (TTM, Tokyo, Japan)	BIA	4-m walk test in 8 m path	AWGS:(low SMI + low HGS) or (low SMI + low gait speed)
	Not clear mentioned	Not clear mentioned	Not clear mentioned	
Sakai et al. (31)	Digital grip strength dynamometer	CC	NA	EWGSOP: low HGS + low CC
	<30 kg for male, <20 kg for female	<34 cm for male; <33 cm for female	NA	

*DEXA, dual-energy X-ray absorptiometry; BIA, bioelectrical impedance analysis; CC, calf circumference; AWGS, Asian Working Group for Sarcopenia; EWGSOP, European Working Group on Sarcopenia in Older People; HGS, hand grip strength, SMI, skeletal muscle mass index*.

*Cuff off points:*

*①, Hand grip strength: male: <26 kg, female: <18 kg*.

*②, Skeletal muscle mass index: male: <7.0 kg/m^2^, female: <5.7 kg/m^2^.*

*③, Skeletal muscle mass index: male: <7.0 kg/m^2^, female: <5.4 kg/m^2^*.

*④, Gait speed <0.8 m/s*.

### Quality Assessment of the Included Studies

The results of the quality assessment are presented in Table 3. The domains for which most studies failed were “enough follow-up period” and “adequacy of follow-up.” This is because the cross-sectional design was employed in the majority of the enrolled articles and the studies did not involve a longitudinal follow-up. The results of the quality assessment are shown in Table 3.

**Table 3:** Quality assessment for the included studies by using the newcastle-ottawa scale.

	**Representative of sarcopenia patients**	**Selection of control**	**Ascertain of sarcopenia measurement**	**Outcome of interest not present at start**	**Comparability of cohorts**	**Assessment of outcome**	**Enough follow-up period**	**Adequacy of follow up**	**Total point**
Shimizu et al. (37)	⋆	⋆	⋆	⋆	⋆⋆	⋆			7
Chen et al. (35)	⋆	⋆	⋆	⋆	⋆⋆	⋆	-	-	7
Kobuchi et al. (36)	⋆	⋆	⋆	⋆	⋆⋆	⋆	-	-	7
Sakai et al. (34)	⋆	⋆	⋆	⋆	⋆⋆	⋆	-	-	7
Wakabayashi et al. (18)	⋆	⋆	⋆	⋆	⋆⋆	⋆	⋆	⋆	9
Kaji et al. (33)	⋆	⋆	⋆	⋆	⋆⋆	⋆	-	-	7
Suzuki et al. (32)	⋆	-	⋆	⋆	⋆⋆	⋆	-	-	6
Ogawa et al. (8)	⋆	⋆	⋆	⋆	⋆⋆	⋆	-	-	7
Machida et al. (30)	⋆	⋆	⋆	⋆	⋆⋆	⋆	-	-	7
Sakai et al. (31)	⋆	⋆	⋆	⋆	⋆⋆	⋆	-	-	7

*⋆, numbers of points earned in each cell*.

### Comparisons of Tongue Pressure Between the Sarcopenic and Non-sarcopenic Group

**Figure 2:**
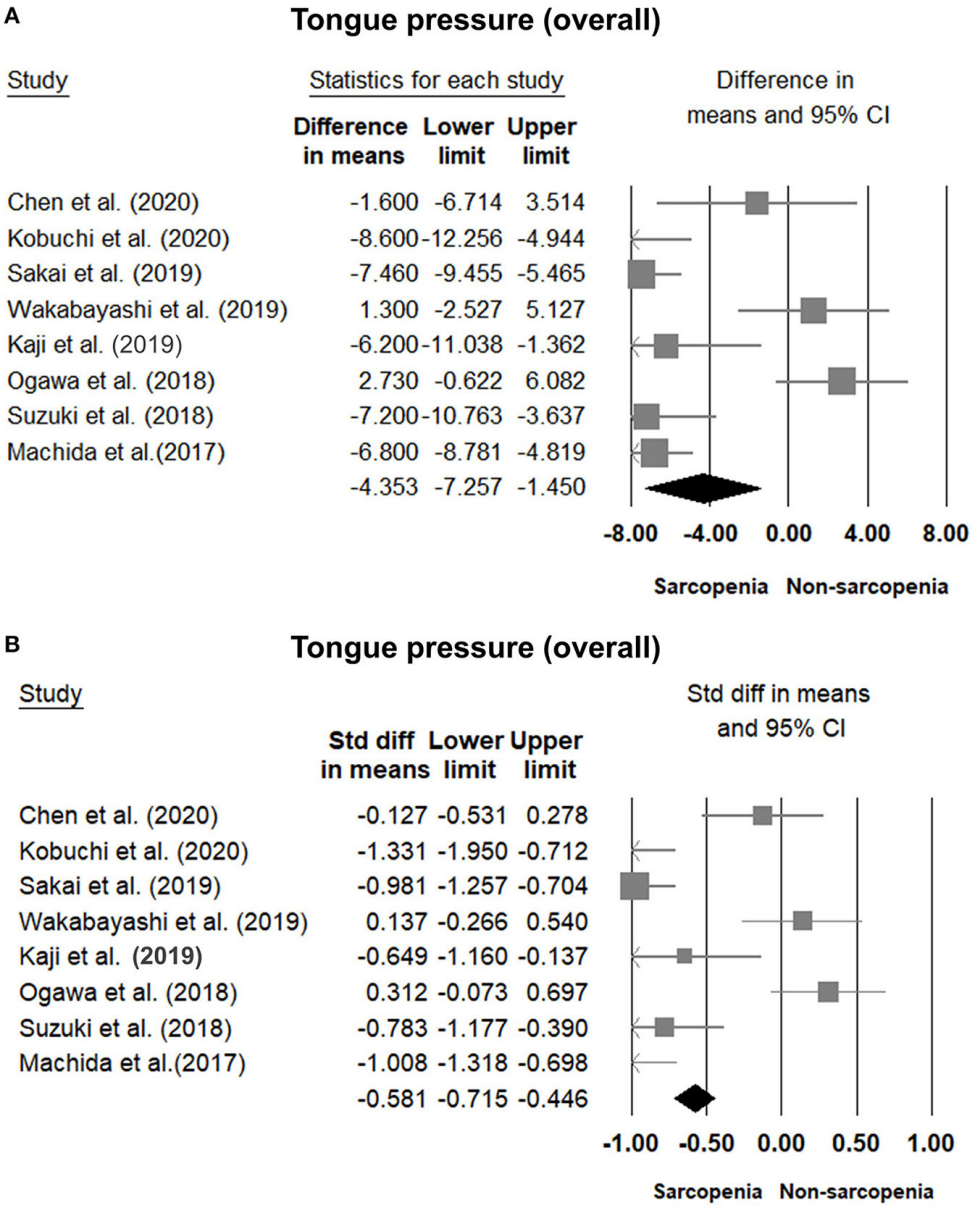
Forest plot of the tongue pressure in the overall participants quantified by the weighted mean difference **(A)** and standardized mean differences **(B)**.

**Figure 3:**
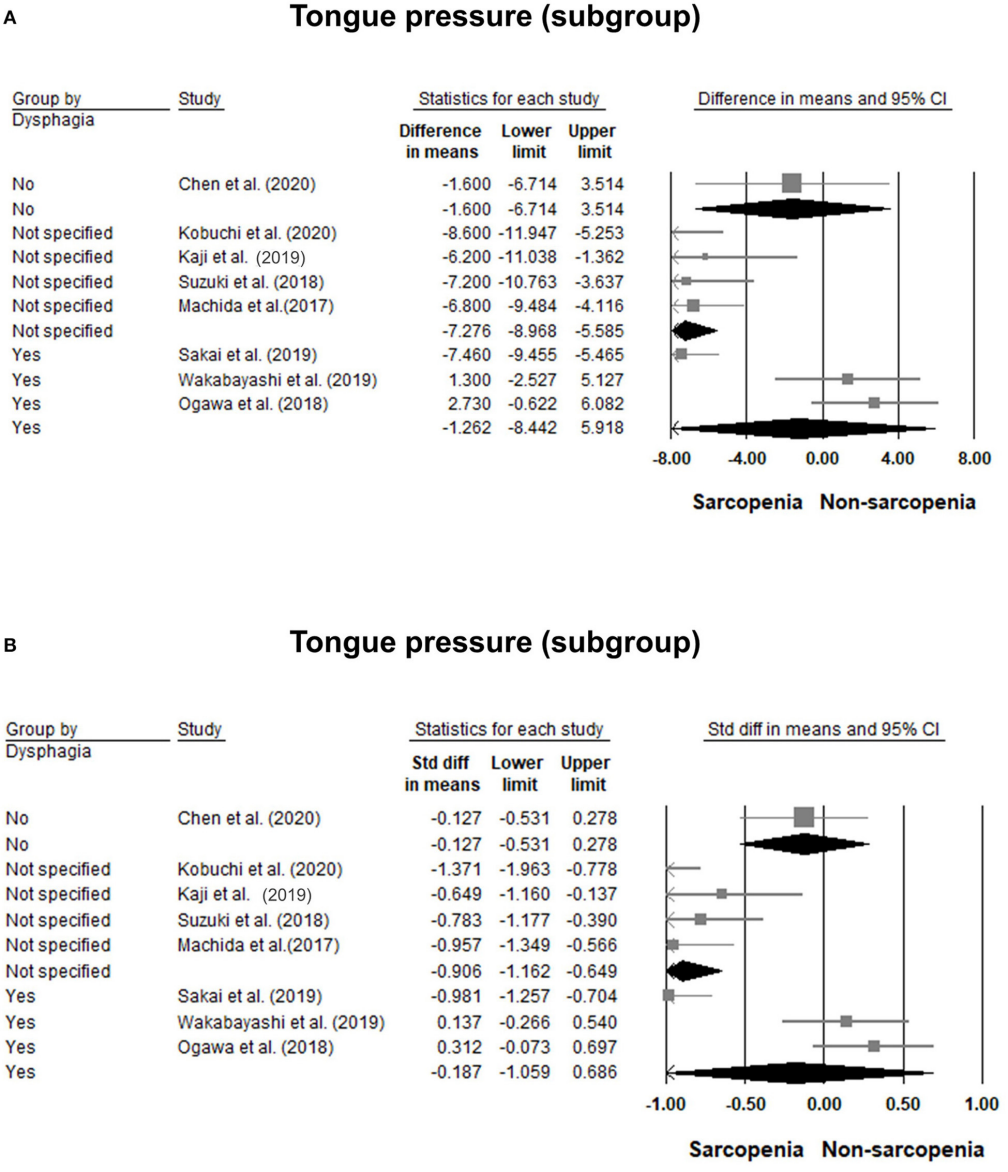
Forest plot of the subgroup analysis of the tongue pressure based on the presence of dysphagia quantified by the weight mean difference **(A)** and standardized mean differences **(B)**.

**Figure 4:**
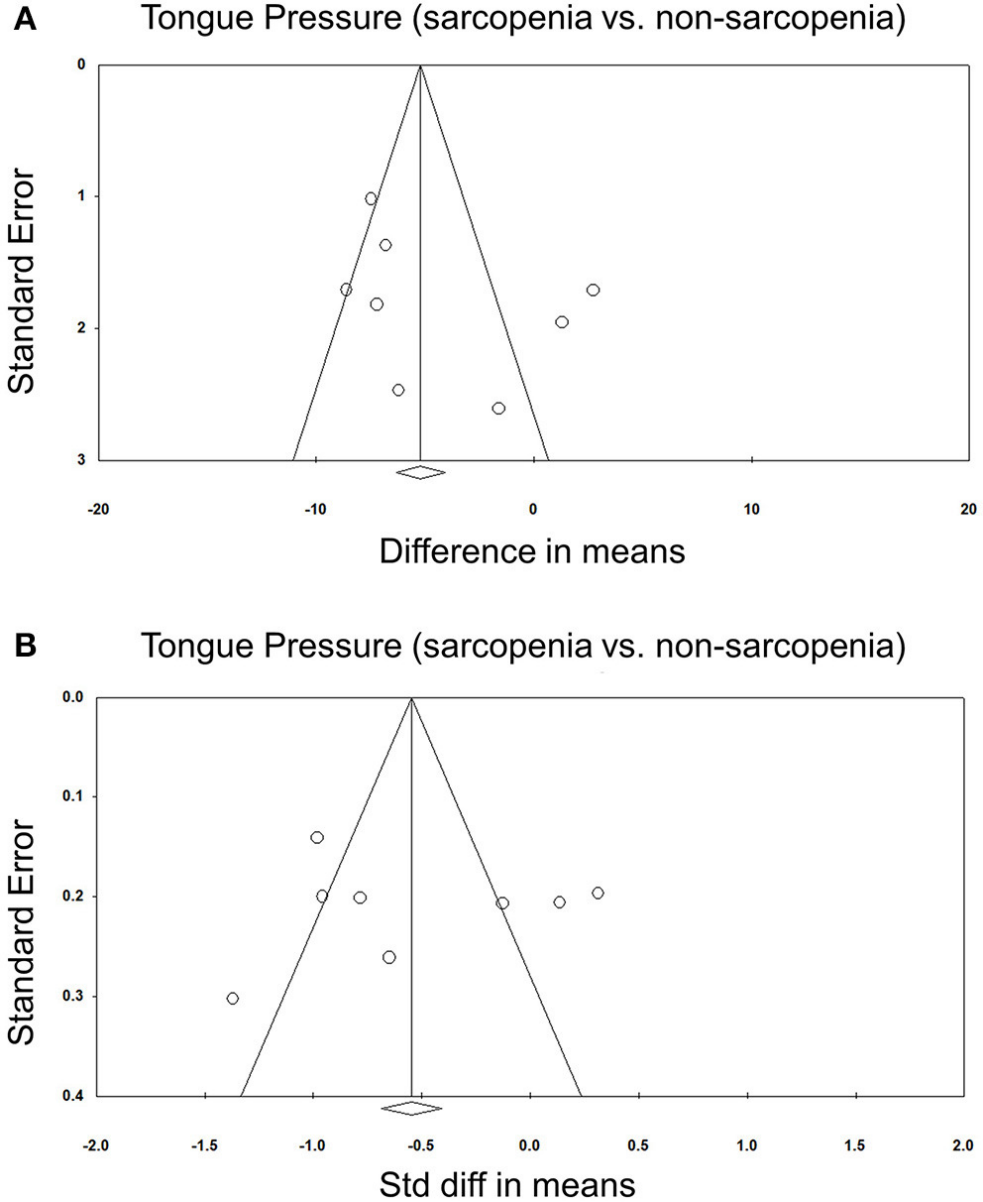
Funnel plot of the weighted mean difference **(A)** and standardized mean differences **(B)** of the tongue pressure between the sarcopenic and non-sarcopenic groups among the included studies. Std diff, standardized difference.

**Figure 5:**
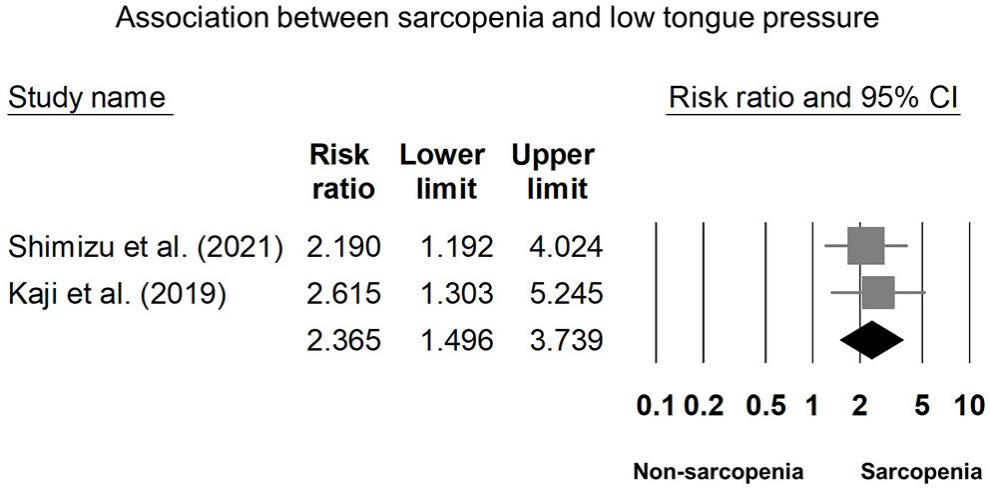
Funnel plot of the association between sarcopenia and low tongue pressure.

Eight of our included studies (8, 18, 30, 32–36) compared tongue pressure. Compared with the non-sarcopenic group, patients with sarcopenia had significantly lower tongue pressure, with a WMD of −4.353 kPa (95% CI, −7.257 to −1.450; *I*^2^ = 84.9%) and an SMD of −0.581 (95% CI, −0.715 to −0.446; *I*^2^ = 88.2%) (Figure 2). Subgroup analysis was performed based on the presence of dysphagia. In studies that recruited patients with dysphagia (8, 18, 34), there was no significant difference in the tongue pressure between the non-sarcopenic and sarcopenic groups, with a WMD of −1.262 kPa (95% CI, −8.442 to 5.918; *I*^2^ = 94.1%) and an SMD of −0.187 (95% CI, −1.059 to 0.686; *I*^2^ = 94.5%). In studies that enrolled patients without specifying whether or not they had dysphagia (30, 32, 33, 36), the sarcopenic group still had significantly lower tongue pressure than the non-sarcopenic group, with a WMD of −7.112 kPa (95% CI, −8.601 to −5.623; *I*^2^ < 0.01%) and an SMD of −0.921 (95% CI, −1.152 to −0.690; *I*^2^ = 15.3%). Only one study included participants without clinical dysphagia (35). The patients in the aforementioned study had a WMD of −1.600 kPa (95% CI, −6.714 to 3.514) and an SMD of −0.127 (95% CI, −0.531 to 0.278) (Figure 3). Visual inspection of the funnel plots and *p*-values following the Egger's test revealed no significant publication bias (Figure 4). The association between sarcopenia and low tongue pressure was available in two (33, 37) of our included studies. The threshold for defining low tongue pressure was 20 kPa in the one conducted by Shimizu et al. (37) and 21.6 kPa in the one conducted by Kaji et al. (33). The pooled analysis indicated that sarcopenia was associated with low tongue pressure, with a risk ratio of 2.365 (95% CI, 1.496 to 3.739; *I*^2^ < 0.001) (Figure 5).

### Comparisons of Tongue Pressure Between Men and Women

**Figure 6:**
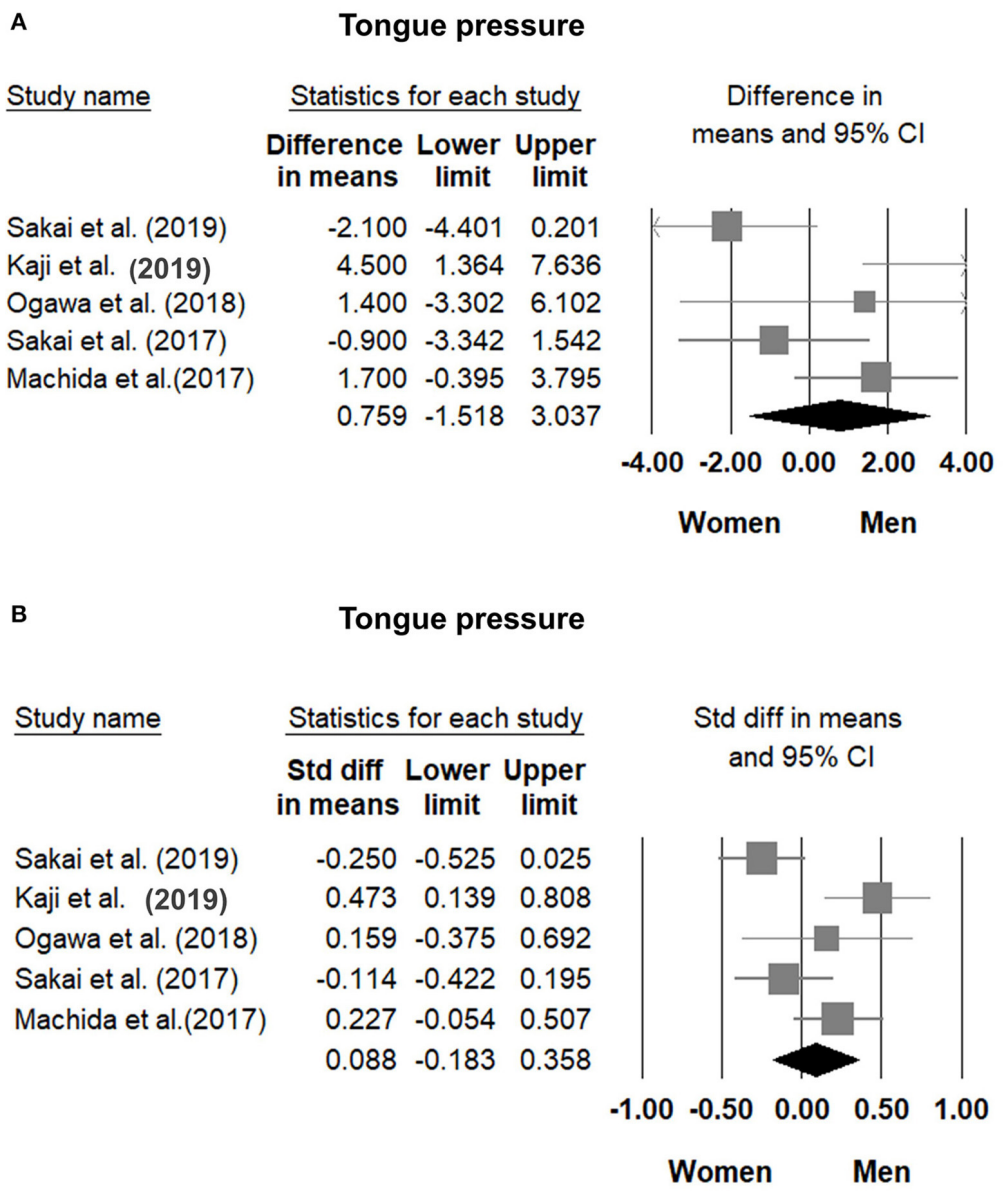
Forest plot of the weighted mean difference **(A)** and standardized mean differences **(B)** of tongue pressure between men and women. Std diff, standardized difference.

**Figure 7:**
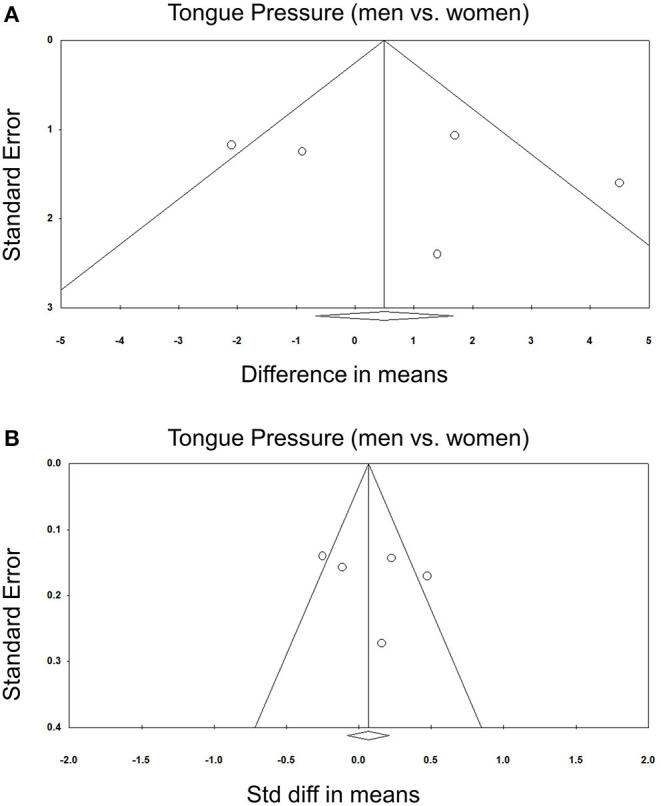
Funnel plot of the weighted mean difference **(A)** and standardized mean differences **(B)** of the tongue pressure between men and women among the included studies. Std diff, standardized difference.

Comparisons of tongue pressure between male and female participants were available in five studies (8, 30, 31, 33, 34). No significant gender differences (men vs. women) were identified. The WMD was 0.759 kPa (95% CI, −1.518 to 3.037; *I*^2^ = 70.0%) and the SMD was 0.088 (95% CI, −0.183 to 0.358; *I*^2^ = 70.2%) (Figure 6). No significant publication bias was detected based on visual inspection of the funnel plots and *p*-values following Egger's test (Figure 7).

### Correlation of Tongue Pressure With Subcomponents of Sarcopenia

**Figure 8:**
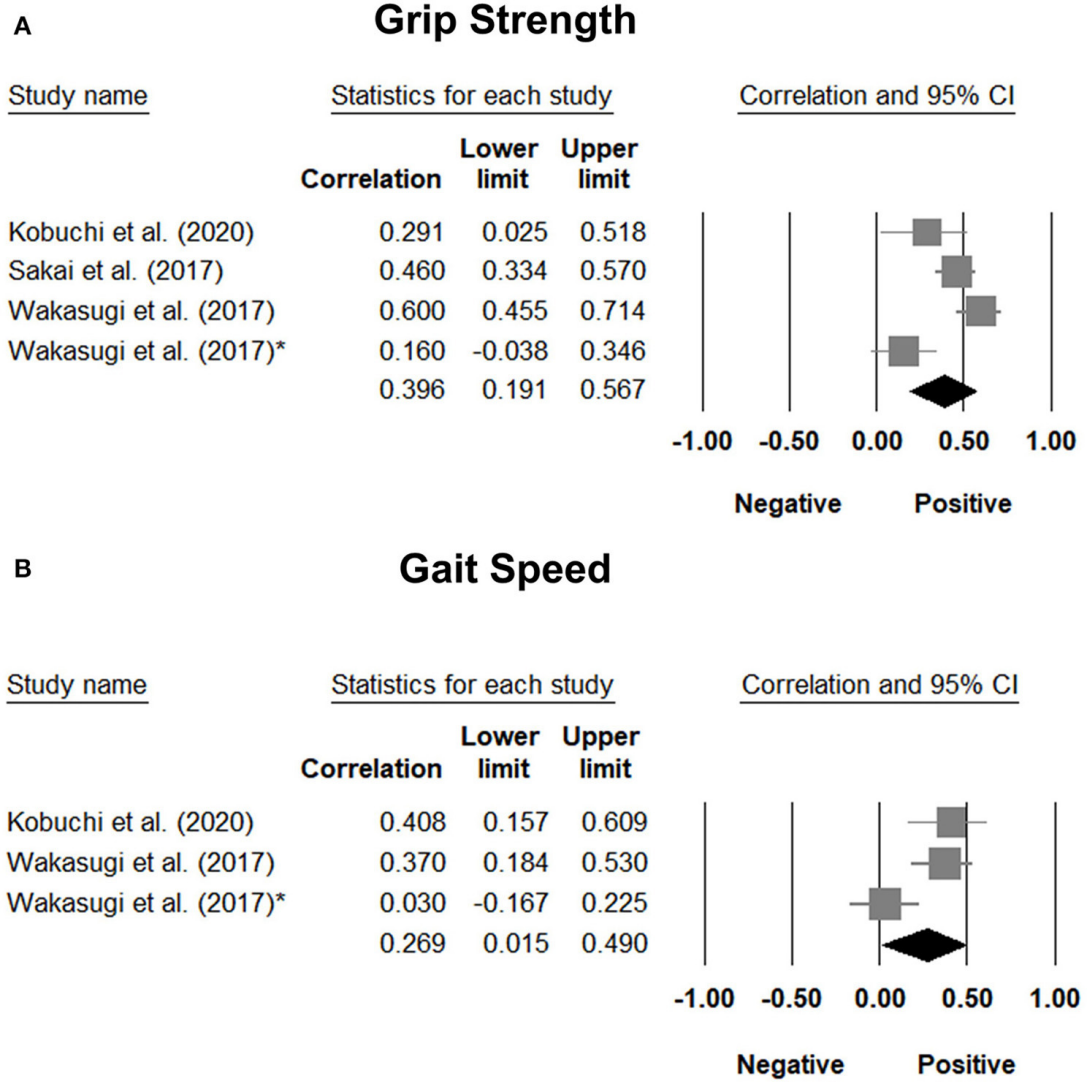
Forest plot of the correlation analysis between tongue pressure and grip strength **(A)** and between tongue pressure and gait speed **(B)**. In the study performed by Wakasugi et al., the correlation analysis was conducted based on different genders. The one without the asterisk is the male subgroup, where as the one with the asterisk is the female subgroup.

The correlation coefficient between tongue pressure and grip strength was available in three studies (29, 31, 36), with a pooled value of 0.396 (95% CI, 0.191 to 0.567). The results of the correlation analysis between tongue pressure and grip strength in the study conducted by Machida et al. (30) was derived from that reported by another study (29), since both studies involved the same population of patients. Two of the included studies (29, 36) reported a correlation coefficient between tongue pressure and gait speed, with a pooled value of 0.269 (95% CI, 0.015 to 0.490) (Figure 8). Likewise, the correlation analysis of tongue pressure and gait speed in the study conducted by Machida et al. (30) was available from another study (29), since both studies involved the same population of patients. The aforementioned analyses indicated a significant positive correlation between tongue pressure, grip strength, and gait speed.

## Discussion

The main finding of this study was that elderly patients with sarcopenia had significantly lower tongue pressure than those without sarcopenia. The subgroup analysis further revealed that there was no significant difference in tongue pressure between patients with sarcopenic dysphagia and those with non-sarcopenic dysphagia. No significant gender differences in tongue pressure were identified in our target population. In addition, a positive association existed between tongue pressure and subcomponents of sarcopenia, including grip strength and gait speed.

In our analysis, patients with sarcopenia had significantly lower tongue pressure, with an SMD of −0.581, indicating a moderate between-group difference. Although the mechanism is not clearly understood, a possible reason is that the generalized decline of muscle mass and strength in the sarcopenic population also affects swallowing-related muscles, such as the tongue, infra-hyoid, supra-hyoid, and pharyngeal muscles. Type II muscle fibers are affected by malnutrition, a potential cause of sarcopenia, more easily than type I muscle fibers (38). Therefore, the swallowing muscles are vulnerable to the effects of insufficient nutrition due to its higher type II fiber content (39). These factors may lead to decreased tongue strength, reduced range of tongue motion, weak contractility of pharyngeal muscle, impaired endurance of swallowing-related muscles, and an increased risk of dysphagia in patients with sarcopenia (13). In addition, a previous study found that tongue-pressure resistance training could improve tongue and supra-hyoid muscle function simultaneously and might be helpful for the prevention of sarcopenic dysphagia (40). Hence, our findings are consistent with existing evidence showing reduced tongue strength in the sarcopenic population.

However, there was no significant difference in tongue pressure between patients with and without sarcopenic dysphagia. We speculated that patients with dysphagia had decreased oral intake, which potentiated disuse atrophy of the tongue muscles regardless of pre-existing sarcopenia. Furthermore, neurological diseases such as stroke (41) and Parkinsonism (42) lead to uncoordinated posterior tongue movement and prolonged tongue elevation (43), which interfere with tongue pressure measurement and subsequent underestimation of tongue strength. Therefore, our findings showed that reduced tongue pressure was not an exclusive indicator of sarcopenic dysphagia.

No gender differences in tongue pressure were identified in our study population. A previous study also revealed no significant gender differences in isometric and peak swallowing pressure measured by intraoral pressure sensors in 20 healthy participants (44). In contrast, some studies have revealed that men have greater maximum tongue pressures than women (45, 46). There were two factors that led to the absence of gender differences in our meta-analysis. First, our study population consisted mainly of older adults, whose tongue strength had already decreased with age. Second, the analysis involved patients with sarcopenia whose tongue strength had also been reduced, based on our analysis. Therefore, since tongue strength declined in our study participants, the gender difference in tongue strength was trivial.

The included studies revealed a positive correlation between tongue pressure, physical performance, and grip strength. Grip strength and physical performance are considered objective measurements of muscle function, a subcomponent of sarcopenia (2, 3). Our findings indicate that sarcopenia is a systemic disease that affects skeletal muscles in the whole body. The decline in tongue strength was shown to be proportional to the impact on the skeletal muscles of the limbs. A previous study reported that nutritional support and rehabilitation exercises to restore physical function could improve sarcopenic dysphagia (47). Therefore, in patients with low sarcopenia subcomponent values or performance, tongue pressure must be examined to detect subclinical dysphagia.

There are several limitations that must be acknowledged. First, the present meta-analysis included a relatively low number of studies. An updated meta-analysis may be needed in the future to include more prospective trials to confirm the association of tongue strength with sarcopenia and sarcopenic dysphagia. Second, all enrolled studies evaluated Asian populations. The generalizability of our findings to other ethnicities requires further validation. Third, nine studies used the AWGS criteria to diagnose sarcopenia (8, 18, 30, 32–37), and one study used the EWGSOP criteria (31). The difference in the diagnostic criteria for sarcopenia led to between-study heterogeneity. Fourth, video-fluoroscopic evaluation of swallowing was not performed in the studies that recruited patients with dysphagia. Therefore, it was difficult to investigate which phase of swallowing was impaired in these patients and how it was related to tongue pressure. Fifth, the majority of the included studies employed a cross-sectional design. Therefore, the causal relationship between sarcopenia and reduced tongue strength was not elucidated in our meta-analysis.

## Conclusion

Based on our meta-analysis, reduced tongue strength is associated with sarcopenia; however, it is not an exclusive marker for sarcopenia. In addition, tongue strength is correlated with the subcomponents of sarcopenia, implying that sarcopenia is a systemic disease that affects the skeletal muscles of the whole body. Therefore, in patients with low sarcopenia subcomponent values or performance, tongue pressure must be examined to detect subclinical dysphagia.

## Data Availability Statement

The original contributions presented in the study are included in the article/Supplementary Material, further inquiries can be directed to the corresponding author/s.

## Author Contributions

K-VC, K-CC, and T-ML conceived and designed the study, recruited the study subjects, and planned and performed the statistical analysis. W-TW, T-GW, D-SH, K-VC, and K-CC contributed to study supervision and critical revision of the manuscript. All authors have read and approved the final manuscript.

## Conflict of Interest

The authors declare that the research was conducted in the absence of any commercial or financial relationships that could be construed as a potential conflict of interest.

## Abbreviations

WMD: Weighted mean difference
SMD: standardized mean difference
MWST: modified water swallowing test
EWGSOP: the European Working Group on Sarcopenia in Older People
AWGS: Asian Working Group for Sarcopenia.

## References

[1] Rosenberg IH. Sarcopenia: origins and clinical relevance. J Nutr. (1997) 127:990S–1S. 10.1093/jn/127.5.990S 9164280

[2] Cruz-Jentoft AJ Baeyens JP Bauer JM Boirie Y Cederholm T Landi F. Sarcopenia: European consensus on definition and diagnosis: Report of the European Working Group on Sarcopenia in Older People. Age Ageing. (2010) 39:412–23. 10.1093/ageing/afq034 20392703 PMC2886201

[3] Chen LK Liu LK Woo J Assantachai P Auyeung TW Bahyah KS. Sarcopenia in Asia: consensus report of the Asian Working Group for Sarcopenia. J Am Med Dir Assoc. (2014) 15:95–101. 10.1016/j.jamda.2013.11.025 24461239

[4] Cruz-Jentoft AJ Landi F Schneider SM Zuniga C Arai H Boirie Y. Prevalence of and interventions for sarcopenia in ageing adults: a systematic review. Report of the International Sarcopenia Initiative (EWGSOP and IWGS). Age Ageing. (2014) 43:748–59. 10.1093/ageing/afu115 25241753 PMC4204661

[5] Janssen I Heymsfield SB Ross R. Low relative skeletal muscle mass (sarcopenia) in older persons is associated with functional impairment and physical disability. J Am Geriatr Soc. (2002) 50:889–96. 10.1046/j.1532-5415.2002.50216.x 12028177

[6] Chang SF Lin PL. Systematic literature review and meta-analysis of the association of sarcopenia with mortality. Worldviews Evid Based Nurs. (2016) 13:153–62. 10.1111/wvn.12147 26844538

[7] Shiozu H Higashijima M Koga T. Association of sarcopenia with swallowing problems, related to nutrition and activities of daily living of elderly individuals. J Phys Ther Sci. (2015) 27:393–6. 10.1589/jpts.27.393 25729176 PMC4339146

[8] Ogawa N Mori T Fujishima I Wakabayashi H Itoda M Kunieda K. Ultrasonography to measure swallowing muscle mass and quality in older patients with sarcopenic dysphagia. J Am Med Dir Assoc. (2018) 19:516–22. 10.1016/j.jamda.2017.11.007 29287693

[9] Dellis S Papadopoulou S Krikonis K Zigras F. Sarcopenic dysphagia. A narrative review. J Frailty Sarcopenia Falls. (2018) 3:1–7. 10.22540/JFSF-03-001 32300688 PMC7155347

[10] White GN O'rourke F Ong BS Cordato DJ Chan DKY. Dysphagia: causes, assessment, treatment, and management. Geriatrics-Us. (2008) 63:15–20. 10.1016/j.clon.2008.03.015 18447407

[11] Fujishima I Fujiu-Kurachi M Arai H Hyodo M Kagaya H Maeda K. Sarcopenia and dysphagia: Position paper by four professional organizations. Geriatr Gerontol Int. (2019) 19:91–7. 10.1111/ggi.13591 30628181

[12] Dodds WJ. Physiology of swallowing. Dysphagia. (1989) 3:171–8. 10.1007/BF02407219 2700955

[13] Robbins J Levine R Wood J Roecker EB Luschei E. Age effects on lingual pressure generation as a risk factor for dysphagia. J Gerontol A Biol Sci Med Sci. (1995) 50:M257–62. 10.1093/gerona/50A.5.M257 7671027

[14] Clark HM Henson PA Barber WD Stierwalt JA Sherrill M. Relationships among subjective and objective measures of tongue strength and oral phase swallowing impairments. Am J Speech Lang Pathol. (2003) 12:40–50. 10.1044/1058-0360(2003/051) 12680812

[15] Yamaguchi A Nasu M Esaki Y Shimada H Yoshiki S. Amyloid deposits in the aged tongue: a postmortem study of 107 individuals over 60 years of age. J Oral Pathol. (1982) 11:237–44. 10.1111/j.1600-0714.1982.tb00161.x 6178810

[16] Konaka K Kondo J Hirota N Tamine K Hori K Ono T. Relationship between tongue pressure and dysphagia in stroke patients. Europ Neurol. (2010) 64:101–7. 10.1159/000315140 20628254

[17] Yoshida M Kikutani T Tsuga K Utanohara Y Hayashi R Akagawa Y. Decreased tongue pressure reflects symptom of dysphagia. Dysphagia. (2006) 21:61–5. 10.1007/s00455-005-9011-6 16544085

[18] WakabayashiHTakahashiRMurakamiT. The prevalence and prognosis of sarcopenic dysphagia in patients who require dysphagia rehabilitation. J Nutr Health Aging. (2019) 23:84–8. 10.1007/s12603-018-1117-230569074

[19] Mori T Fujishima I Wakabayashi H Oshima F Itoda M Kunieda K. Development, reliability, and validity of a diagnostic algorithm for sarcopenic dysphagia. JCSM Clin Rep. (2017) 2:10. 10.17987/jcsm-cr.v2i2.17

[20] Suzuki M Kimura Y Otobe Y Kikuchi T Masuda H Taguchi R. Relationship between sarcopenia and swallowing capacity in community-dwelling older women. Gerontology. (2020) 66:549–52. 10.1159/000511359 33075773

[21] Moher D Liberati A Tetzlaff J Altman DG Group P. Preferred reporting items for systematic reviews and meta-analyses: the PRISMA statement. J Clin Epidemiol. (2009) 62:1006–12. 10.1016/j.jclinepi.2009.06.005 19631508

[22] Stang A. Critical evaluation of the Newcastle-Ottawa scale for the assessment of the quality of nonrandomized studies in meta-analyses. Eur J Epidemiol. (2010) 25:603–5. 10.1007/s10654-010-9491-z 20652370

[23] Chiu YH Chang KV Chen IJ Wu WT özçakar L. Utility of sonoelastography for the evaluation of rotator cuff tendon and pertinent disorders: a systematic review and meta-analysis. Eur Radiol. (2020) 30:6663–72. 10.1007/s00330-020-07059-2 32666319

[24] Cohen J. Statistical Power Analysis for the Behavioral Sciences. 2. New Jersey: Lawrence Erlbaum Associates (1988).

[25] Viechtbauer W. Conducting Meta-Analyses in R with the metafor Package. J. Statist. Softw. (2010) 36:1–48. 10.18637/jss.v036.i03

[26] Wu WT Lee TM Han DS Chang KV. The prevalence of sarcopenia and its impact on clinical outcomes in lumbar degenerative spine disease-a systematic review and meta-analysis. J Clin Med. (2021) 10:773. 10.3390/jcm10040773 33671958 PMC7919040

[27] Higgins JP Thompson SG Deeks JJ Altman DG. Measuring inconsistency in meta-analyses. BMJ. (2003) 327:557–60. 10.1136/bmj.327.7414.557 12958120 PMC192859

[28] Lin L Chu H. Quantifying publication bias in meta-analysis. Biometrics. (2018) 74:785–94. 10.1111/biom.12817 29141096 PMC5953768

[29] Wakasugi Y Tohara H Machida N Nakane A Minakuchi S. Can grip strength and/or walking speed be simple indicators of the deterioration in tongue pressure and jaw opening force in older individuals? Gerodontology. (2017) 34:455–9. 10.1111/ger.12292 28836306

[30] Machida N Tohara H Hara K Kumakura A Wakasugi Y Nakane A. Effects of aging and sarcopenia on tongue pressure and jaw-opening force. Geriatr Gerontol Int. (2017) 17:295–301. 10.1111/ggi.12715 26800427

[31] Sakai K Nakayama E Tohara H Maeda T Sugimoto M Takehisa T. Tongue strength is associated with grip strength and nutritional status in older adult inpatients of a rehabilitation hospital. Dysphagia. (2017) 32:241–9. 10.1007/s00455-016-9751-5 27687521

[32] Suzuki M Koyama S Kimura Y Ishiyama D Otobe Y Nishio N. Relationship between characteristics of skeletal muscle and oral function in community-dwelling older women. Arch Gerontol Geriatr. (2018) 79:171–5. 10.1016/j.archger.2018.09.003 30265912

[33] Kaji A Hashimoto Y Kobayashi Y Sakai R Okamura T Miki A. Sarcopenia is associated with tongue pressure in older patients with type 2 diabetes: a cross-sectional study of the KAMOGAWA-DM cohort study. Geriatr Gerontol Int. (2019) 19:153–8. 10.1111/ggi.13577 30585390

[34] Sakai K Nakayama E Tohara H Takahashi O Ohnishi S Tsuzuki H. Diagnostic accuracy of lip force and tongue strength for sarcopenic dysphagia in older inpatients: A cross-sectional observational study. Clin Nutr. (2019) 38:303–9. 10.1016/j.clnu.2018.01.016 29398338

[35] Chen YC Chen PY Wang YC Wang TG Han DS. Decreased swallowing function in the sarcopenic elderly without clinical dysphagia: a cross-sectional study. BMC Geriatr. (2020) 20:419. 10.1186/s12877-020-01832-0 33087067 PMC7579958

[36] Kobuchi R Okuno K Kusunoki T Inoue T Takahashi K. The relationship between sarcopenia and oral sarcopenia in elderly people. J Oral Rehabil. (2020) 47:636–42. 10.1111/joor.12948 32072652

[37] Shimizu A Maeda K Nagami S Nagano A Yamada Y Shimizu M. Low tongue strength is associated with oral and cough-related abnormalities in older inpatients. Nutrition. (2021) 83:111062. 10.1016/j.nut.2020.111062 33348111

[38] Hortobagyi T Zheng D Weidner M Lambert NJ Westbrook S Houmard JA. The influence of aging on muscle strength and muscle fiber characteristics with special reference to eccentric strength. J Gerontol A Biol Sci Med Sci. (1995) 50:B399–406. 10.1093/gerona/50A.6.B399 7583797

[39] Mccausland T Bordoni B. Anatomy, Head and Neck, Genioglossus Muscle. Treasure Island, FL: StatPearls (2021). 31424725

[40] Namiki C Hara K Tohara H Kobayashi K Chantaramanee A Nakagawa K. Tongue-pressure resistance training improves tongue and suprahyoid muscle functions simultaneously. Clin Interv Aging. (2019) 14:601–8. 10.2147/CIA.S194808 30962680 PMC6432900

[41] Lee JH Kim HS Yun DH Chon J Han YJ Yoo SD. The relationship between tongue pressure and oral dysphagia in stroke patients. Ann. Rehab. Med. Arm. (2016) 40:620–8. 10.5535/arm.2016.40.4.620 27606268 PMC5012973

[42] Minagi Y Ono T Hori K Fujiwara S Tokuda Y Murakami K. Relationships between dysphagia and tongue pressure during swallowing in Parkinson's disease patients. J. Oral Rehabil. (2018) 45:459–66. 10.1111/joor.12626 29575051

[43] Blonsky ER Logemann JA Boshes B Fisher HB. Comparison of speech and swallowing function in patients with tremor disorders and in normal geriatric patients: a cinefluorographic study. J Gerontol. (1975) 30:299–303. 10.1093/geronj/30.3.299 1120891

[44] Nicosia MA Hind JA Roecker EB Carnes M Doyle J Dengel GA. Age effects on the temporal evolution of isometric and swallowing pressure. J Gerontol A Biol Sci Med Sci. (2000) 55:M634–40. 10.1093/gerona/55.11.M634 11078092

[45] Youmans SR Stierwalt JA. Measures of tongue function related to normal swallowing. Dysphagia. (2006) 21:102–11. 10.1007/s00455-006-9013-z 16685469

[46] Stierwalt JA Youmans SR. Tongue measures in individuals with normal and impaired swallowing. Am J Speech Lang Pathol. (2007) 16:148–56. 10.1044/1058-0360(2007/019) 17456893

[47] Kasahara K Okubo K Morikawa J. Laryngeal suspension, combined with rehabilitation and nutritional support, improved the clinical course of a patient with sarcopenic dysphasia. Int J Surg Case Rep. (2020) 70:140–4. 10.1016/j.ijscr.2020.04.068 32416483 PMC7229273

