# Supplementary material for: Assessment of Tongue Strength in Sarcopenia and Sarcopenic Dysphagia: A Systematic Review and Meta-Analysis
Source: Front Nutr. 2021 Jun 24;8:684840. doi: 10.3389/fnut.2021.684840 (PMC8264147; doi:10.3389/fnut.2021.684840)
Supplement: Supplementary file 1 [file Data_Sheet_1.DOCX]

**Appendix 1. Strategy of Literature Search**

**PubMed**

1. (‘sarcopenia’ OR ‘frailty’)
2. (‘dysphagia’ OR ‘swallowing disorder’)
3. (‘tongue pressure’ OR ‘tongue strength’).
4. 1 and 2 and 3

**Embase**

1. (‘sarcopenia’ OR ‘frailty’)
2. (‘dysphagia’ OR ‘swallowing disorder’)
3. (‘tongue pressure’ OR ‘tongue strength’).
4. 1 and 2 and 3
